# Presence of *Helicobacter pylori *in a Mexican Pre-Columbian Mummy

**DOI:** 10.1186/1471-2180-8-119

**Published:** 2008-07-15

**Authors:** Gonzalo Castillo-Rojas, Marco A Cerbón, Yolanda López-Vidal

**Affiliations:** 1 Programa de Inmunología Molecular Microbiana, Departamento de Microbiología y Parasitología. Facultad de Medicina, Universidad Nacional Autónoma de México, Mexico City, Mexico; 2 Departamento de Biología. Facultad de Química, Universidad Nacional Autónoma de México, Mexico City, Mexico

## Abstract

**Background:**

Recent studies showed that *Helicobacter pylori *existed in the New World prior to the arrival of Columbus. The purpose of the present study was to detect the presence of *Helicobacter pylori *in pre-Columbian mummies from Northern Mexico.

**Methods:**

Six samples were studied (four samples of gastric remains, tongue-soft palate, and brain remained as negative controls) from two of the six naturally mummified corpses studied (adult male and infant male). Samples were taken from tissues suitable for DNA amplification by Polymerase chain reaction (PCR). DNA was extracted and *H. pylori *detection was carried out by PCR and hybridized with the pHp probe from *16S rRNA *gene. The purified PCR products were cloned and sequenced in both directions. DNA sequences were analyzed with ALIGN and BLAST software. A second amplification was performed using *ureB *gene by real-time PCR.

**Results:**

From four samples of gastric remnant, only two were *H. pylori-*positive for amplification of a 109 bp DNA fragment; the remaining two were negative, as were the tongue-soft palate and the brain biopsies as well. These PCR products were hybridized with a pHp probe. Nucleotide sequence analysis showed homology with *H. pylori *in 98 of 99% when compared with the gene bank nucleotide sequence. Only one sample of gastric remnant *H. pylori*-positive with *16S rRNA *gene was also positive for *ureB *gene from *H. pylori*.

**Conclusion:**

This data supported infection with *H. pylori *in Mexican pre-Columbian mummies dating from approximately 1,350 AC.

## Background

*Helicobacter pylori *(*H. pylori*) microaerophilic Gram-negative bacteria, which colonize the human stomach, are associated with an increased risk of developing gastric cancer and peptic ulcer disease [1]. In a study on the survival of antigenic material in mummified human remains from the Andean area of South America, Allison *et al*. found that fecal specimens harbored antigens from *H. pylori *nearly 3,000 years old [2]. Several studies based on genotypic analysis of *H. pylori *strains isolated from Latin-American patients exhibited European genotypes [3,4]. Kersulyte *et al*. have suggested that *H. pylori *may have been brought to the New World by European conquerors and colonist's ca 500 years ago, because the authors found a great similarity among predominant genotypes in Europe and South America [5]. However, Ghose *et al*. found evidence that the ancestors of present-day Amerindians carried *H. pylori *(in the stomach) when they migrated from Asia at least 11,000 years ago across the Bering Strait [6]. These data are consistent with that found by Yamaoka *et al*. when they studied isolated *H. pylori *strains from Amerindians from Colombia who had scarce or nil contact with Europeans [7]. On the other hand, Falush *et al*. and Linz *et al*. studied *H. pylori *isolates from diverse ethnic sources. These authors assigned to *H. pylori *strains into seven population and sub-populations clusters with different geographical distributions and suggested that these modern populations derive their gene pools from ancestral populations that arose in Africa, Central Asia, and East Asia. This suggesting that subsequent spread could be attributed to human migratory fluxes such as the prehistoric colonization of Polynesia and the Americas [8,9].

Physical anthropologists at the Instituto Nacional de Antropología e Historia (INAH, Mexico) are in charge of the registration, preservation, and study of mummies (including offerings and funerary apparel) discovered in the country. The majority of Mexican mummies are found in dry places such as caves or rock shelters, where rapid dehydration occurred. Mummification is defined as the preservation of soft weaves in the enzymatic process of postmortem putrefaction; this can take place by means of environmental effects, or as a result of human intervention. In Mexico, mummies are a product of natural dryness process exerted by one or various factors (climate, temperature, place of deposit, funerary trousseau, etc.). In the case of the funeral caves located in Northern Mexico, the hot, dry climate is favorable for organic preservation. On the other hand, as part of the funerary rite bodies were wrapped in blankets woven from absorbent vegetable fibers and/or in *petates *(sleeping mats), conforming funeral lumps. The internal organs are the last to dehydrate; thus, these are susceptible to putrefaction and are seldom conserved. The purpose of the present study was to detect the presence of *H. pylori *in pre-Columbian mummies of Northern Mexico.

## Methods

**Figure 1:**
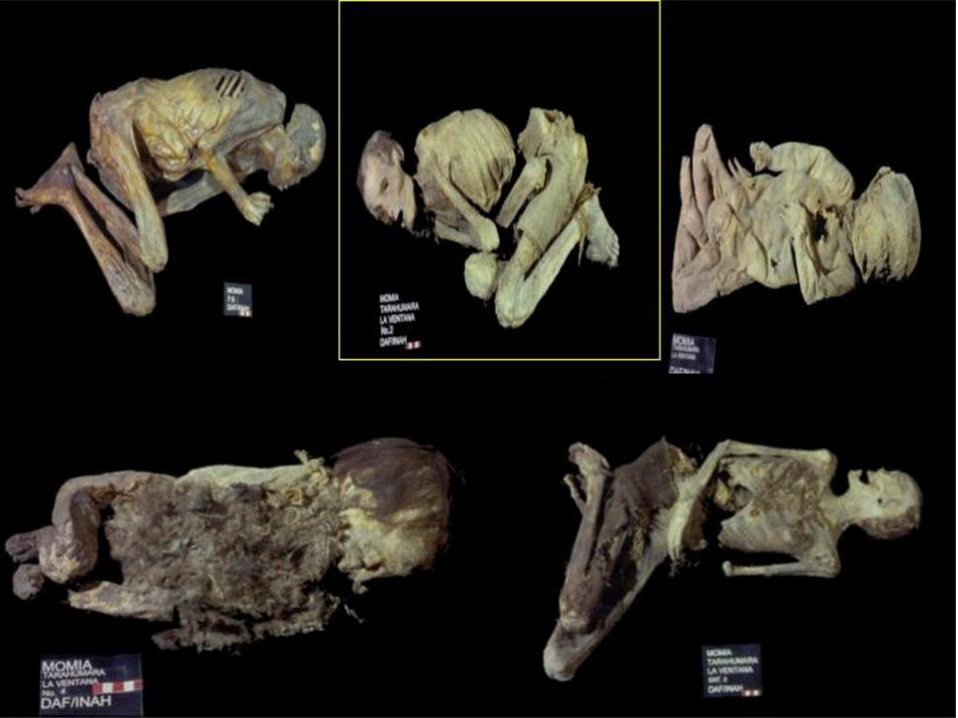
Mexican Pre-Columbian mummies. Yellow box showed the mummy that was *Helicobacter pylori*-positive.

Four pre-Columbian mummified bodies recovered in the funeral cave of *La Ventana*, located in the Chihuahua State desert and two additional mummies recovered in a cave located in the state of Durango (Figure 1) were studied. Climatic conditions in the caves allowed natural preservation of the bodies throughout centuries. (We are grateful to Dr. Josefina Mansilla at Instituto de Antropología e Historia de México who allowed us to obtain mummy samples). The samples were studied to obtain valuable information on the soft tissues and for internal-organ visualization using a Pentax EG-1840 gastroscope, a Pentax VB-1530 videobronchoscope, and a Pentax ELC-2800 videolaparoscope. (We kindly appreciate the support from Dr. Fernando Mundo of the Hospital Angeles del Pedregal in Mexico City for sample collection). Sampling was carried out in a room with no history of work with *H. pylori *at the Instituto de Antropología e Historia de México. Tissue specimens from the mummies were acquired by dissection and placed into sterile plastic containers. Aseptic techniques were employed for sampling these specimens to avoid contamination with either contemporary *H. pylori *DNA or cross-contamination among samples. Four tissue samples of gastric remains, tongue-soft palate, and brain were obtained from only two mummies, in which the natural orifices found in the mummies allowed exploration of internal structures without damaging tissues or fabrics surrounding the corpses (Table 1).

**Table 1:** Study areas in Mexican Pre-Columbian mummies

Mummy	Sex	Age	Head Neck	Thorax	Abdomen	Biopsy
1	M	Infant	Yes	Yes	Yes	-
**2**	**M**	**Adult**	**Yes**	**Yes**	**Yes**	**Yes**
**3**	**M**	**Infant**	**Yes**	**Yes**	**Yes**	**Yes**
4	M	Infant	Yes	Yes	Yes	-
5	F	Infant	Yes	Yes	Yes	-

DNA extraction was conducted in another room with no history of work with *H. pylori *and was performed with GeneClean for Ancient DNA kit (BIO 101, CA, USA) employing sterile disposable labware; gloves were worn at all times and were always changed prior to proceeding from one area to another. *H. pylori 16S rRNA *gene detection was performed by Polymerase chain reaction (PCR). These assays were handled in three separated areas: one for set-up; one for the PCR itself, and one for work-up of the reactions. These PCR products were hybridized with the pHp probe according to Castillo-Rojas *et al*. [10]. This double-blind experiment was carried out at a second laboratory that was unrelated with *H. pylori *assaying. The purified PCR products were cloned in a pCR 2.1-TOPO vector (Invitrogen, Life Technologies) and sequenced in both directions using an ABI PRISM, BigDye Terminator Cycle Sequencing Ready Reaction Kit and the ABI PRISM 377 DNA Sequencer (PE Biosystems). DNA sequences were analyzed with ALIGN (Scientific and Educational software) and BLAST 2.0 (Basic Local Alignment Search Tool) software [11].

On the other hand, *H. pylori ureB *gene detection was performed by real-time PCR, primers and Taqman probe were designed for amplify a conserved region from the *H. pylori ureB *gene (manuscript in preparation); this assay was handled in two separated areas: one for set-up and one for the PCR itself.

The tissue sample from mummy 2 was hydrated in sterile and filtrated phosphate buffered saline; the sample was immersed in multiple baths of progressively concentrated ethanol and then immersed in xylene. Finally, the sample was embedded in hot molten paraffin wax and placed in a mold containing additional molten wax, allowing this to cool and harden. The tissue was then sectioned into very thin (5-micrometer) sections with a microtome and placed on a glass slide for staining with hematoxylin and eosin [12].

## Results

**Figure 2:**
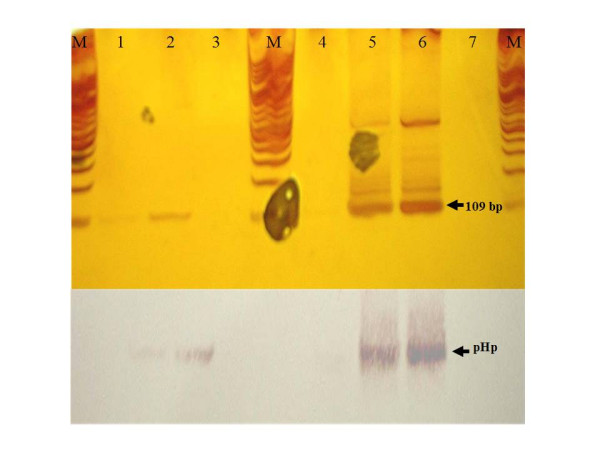
Polymerase chain reaction (PCR) amplification and hybridization of biopsy samples. **A**) PCR products of the 109-bp fragment of *16S rRNA *obtained from *H. pylori*. Line: 1) gastric remains I; 2) gastric remains II; 3) tongue-soft palate; 4) brain; 5) *H. pylori *ATCC 43504; 6) *H. pylori *84–183, and 7) negative control reaction. M = Molecular weight marker (100-bp DNA ladder). **B**) Hybridization of PCR products with pHp probe.

Of four biopsies of gastric remains, only two were *H. pylori-*positive for amplification of a 109-bp DNA fragment (from mummy 2); the remaining two gastric remains-tissue samples were *H. pylori- *negative, as were the tongue-soft palate and brain-tissue samples (Figure 2A). PCR products were *H. pylori-*positive to hybridization with *H. pylori *probe (pHp) (Figure 2B), confirming that the amplified product of 109-bp is *H. pylori*-specific. The results of sample amplification and hybridization performed at the second laboratory was in agreement with our results: only gastric-remains samples from mummy 2 were positive, while the other remaining samples of gastric remains, in addition to tongue-soft palate and brain, were also negative.

**Figure 3:**
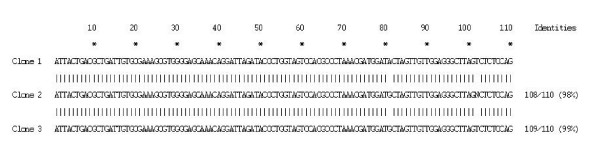
Sequence alignment of the *16S rRNA *gene obtained from three clones of mummy 2.

**Figure 4:**
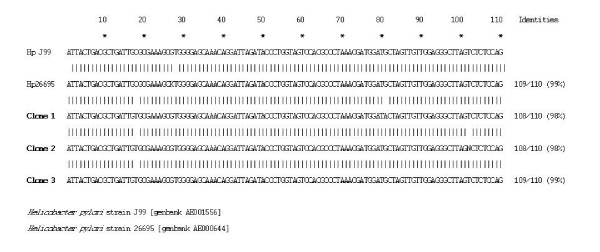
Sequence alignment of *16S rRNA *gene obtained from mummy 2 compared with *H. pylori *strain using BLASTN.

Analysis of nucleotide sequences among the three clones sequenced showed a homology of 98 and 99% (Figure 3). On comparison with *H. pylori *26695 and J99 nucleotide sequences using BLAST, these sequences showed a homology of 98–99% (Figure 4).

**Figure 5:**
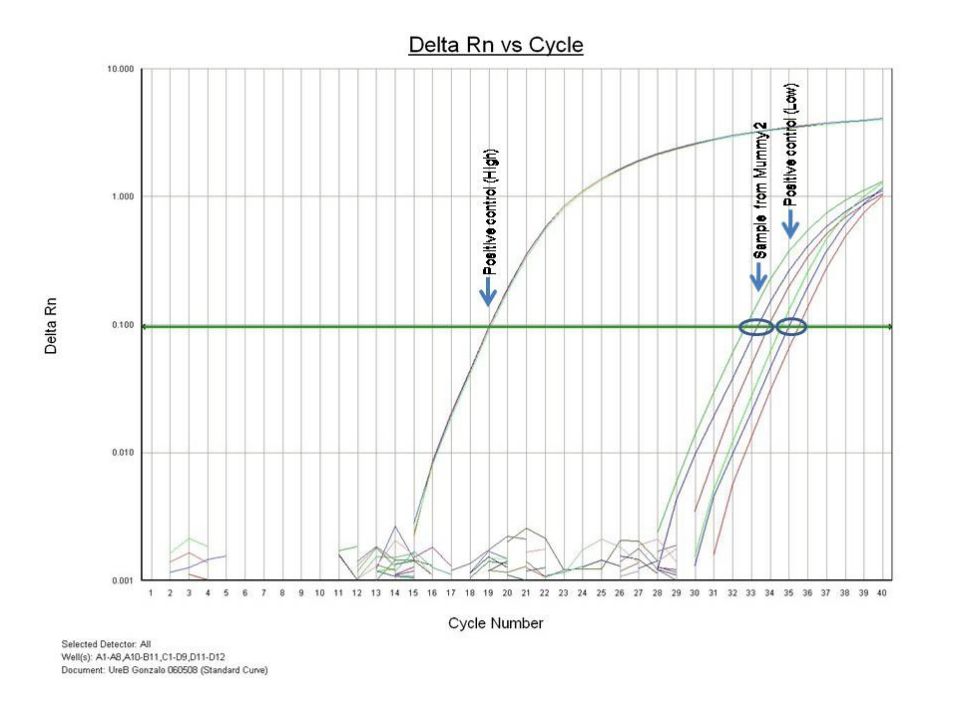
*ureB *gene detection of *H. pylori *by real-time PCR using Taqman probe. Control positive are a fragment of 97 bp from *ureB *gene of *H. pylori *26695 (ATCC 700392) cloned in pPCR-TOPO 2.1 (Invitrogen). High and low positive control equivalent to approximately 6 × 10^6 ^and 60 copies of the *ureB *gene, respectively.

*ureB *gene detection by real-time PCR showed that only one of the two samples *H. pylori-*positive(for amplification of *16S rRNA *gene) was also positive (Figure 5).

**Figure 6:**
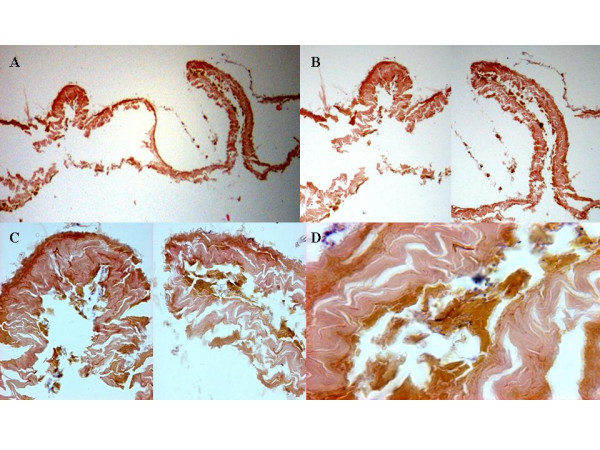
Histological examinations of gastric remains of a Mexican mummy with hematoxin-eosin stain. A) 4×; B) 10×; C) 40×, and D) 100×.

Histological analysis of the gastric remains showed the presence of a structure resembling simple columnar epithelium followed by an invagination attached to two structures resembling bands similar to cords, which were also made up of simple columnar tissue. This image probably represents the union of gastric tissue with large intestine tissue, because the structure observed presented a resemblance with intestine villus (Figures 6A and 6B). We observed tissue loss in the remainder of the samples and lack of the presence of lamina propria. Some areas of tissue observed resembled the submucosa (Figure 6C) and we observed no type of inflammatory processes in the tissue (Figure 6D).

## Discussion

The study of mummified remains is intended to increase knowledge about human beings and their diseases by relating the pathological findings in tissues, which preserve historical and sociocultural knowledge. Some diseases may be diagnosed in ancient skeletons, but a larger number could be recognized by the study of soft mummified tissues [13]. Paleopathology is based on anatomic, immunohistological, and genetic studies. Imaging methods and endoscopic procedures provide valuable data when direct examination is not possible. Imagenology supplies important anthropological and medical information (for example, contents of the mortuary bundle, presence of skeletal deformations, etc.) [14]. Flexible endoscopes may be inserted through natural orifices, through orifices caused by tissue deterioration, and even with the aid of laparoscopic drills, for direct observation of organic structures and/or sampling [15].

DNA begins to chemically fragment and change after death; however, there are small fragments that may be preserved for centuries, which may be amplified and identified by means of PCR; the DNA of infectious agents can also be amplified and detected by PCR. Due to its conservational properties, the *16S *bacterial ribosomal DNA subunit allows genus and species identification of the genus and species. In this manner, identification of genic segments of *Mycobacterium tuberculosis *[16,17], *Mycobacterium leprae *[18], *Treponema pallidum *[19], *Trypanosoma cruz *[20,21], intestinal bacteria, and human T-cell lymphotropic virus type [22] have been identified in mummified human remains.

In a recent review, A.C. Aufderheid noted that endoscopic procedures in thoracic and abdominal cavities may be ineffective due to that the pleural adhesion may have poor visibility and the abdominal cavity is often obliterated by the collapsed, rigid, and dry abdominal wall in non-eviscerated mummified bodies [23]. Furthermore, Aufderheid mentioned that biopsy tweezers for clinical endoscopes are not designed to handle the dry, hard, and brittle tissues of mummified bodies. In our study, endoscopic procedures were performed without technical handicaps, and we were able to study the abdominal and thoracic cavities, as well as to visualize the organic remains, efficiently. It is noteworthy that in one case, the endoscope was introduced through a defect in the dorsal vertebra and explored the medullar channel up to the point at which it meets the cranial bones. Conventional clinical forceps were successfully utilized to foreign-body extraction and for sampling.

Pelayo-Correa *et al*. and Allison *et al*. found evidence of *H. pylori *in coprolites from two Andean mummies dating from 300 AD; these authors concluded that their report constitutes the oldest evidence of infection in humans by *H. pylori *[2,24]. The pathogenic role of the bacteria is established if the microbe causes a disease or even if it is only carried. In the case of *H. pylori*, infection is established when the microorganism infiltrates the stomach lining and induces a local inflammatory response, unlike colonization, which does not cause an inflammatory response and which takes place in the digestive tract. Therefore, the findings of Pelayo-Correa and Allison are not necessarily indicative of *H. pylori *infection because they solely detected specific protein. In our work, detection of genetic material of *H. pylori *directly in gastric tissue supported the pathogenic role of the bacteria in humans from ancestral times.

With regard to the origin of *H. pylori*, our results support the Asiatic provenance of this bacterium, because paleopathologic evidence studied in mummies strongly suggest their pre-Hispanic origin. The results of this study are not intended to put an end to the controversy regarding the Asiatic or European origin [5,6], for this would require genotypic identification of ancestral *H. pylori *and comparison with prevalent types in Europe and Asia [4,8,9], as, for example, that the *vacA *gene's slc allele is isolated only in Asiatic individuals [25].

In mummy 2, we documented by x-rays the presence of vertebral changes as a type of deforming spondylosis, a degenerative disease of the axial skeleton characterized by formation of new bone in anterior and lateral segments of vertebrate bodies associated with minimal alteration of the intervertebral disk. Bone excrescencies, characteristic of this condition, have been associated with activities requiring considerable physical effort, and its prevalence increases from the sixth decade of life [26]. Pre-Hispanic peoples from the northern region of Mexico were nomadic tribes devoted mainly to hunting and agriculture, which presumably accounts for chronic trauma of the axial skeleton.

In conclusion, only mummy 2 was positive to *H. pylori*, which was present in colon remnant material. This male mummy has a radiographic age between 50 and 60 years; this mummy has been radiocarbon-dated at approximately 1,000–1,400 AD or 950-550 calendar years before the present time. Furthermore, on considering geographical location, disposition, plumage, textiles, clothing, funerary apparel, and ceramic technique [14], we estimate that these mummies belong to the Pre-Columbian period, circa 1,350 AD. Nevertheless, contemporary contamination cannot be discarded. On the other hand, because skeletal information is a limited source for paleopathological studies due to that not all diseases leave marks on bones, videoendoscopic documentation and sampling can provide an important resource for study of the diseases and lifestyles of early Native American groups.

## Authors' contributions

GCR performed amplification, hybridization, and sequencing of the DNA fragment for *H. pylori*. MAC conducted repeat in bind for *H. pylori *amplification and hybridization at a second laboratory not related with *H. pylori *assaying. YLV performed experimental strategy, the writing and discussion of this manuscript.

## Conflict of interest statement

The authors declare that they have no competing interests.
